# Supplementary material for: B cell analyses after SARS-CoV-2 mRNA third vaccination reveals a hybrid immunity like antibody response
Source: Nat Commun. 2023 Jan 4;14:53. doi: 10.1038/s41467-022-35781-6 (PMC9811867; doi:10.1038/s41467-022-35781-6)
Supplement: Supplementary file 1 — Supplementary Information [file 41467_2022_35781_MOESM1_ESM.pdf]

Supplementary information

B cell analyses after SARS-CoV-2 mRNA third vaccination reveals a hybrid immunity like antibody response

Emanuele Andreano[1,†], Ida Paciello[1,†], Giulio Pierleoni[2], Giulia Piccini[3], Valentina Abbiento[1], Giada Antonelli[1], Piero Pileri[1], Noemi Manganaro[1], Elisa Pantano[1], Giuseppe Maccari[4], Silvia Marchese[5], Lorena Donnici[6], Linda Benincasa[2], Ginevra Giglioli[2], Margherita Leonardi[2,3], Concetta De Santi[1], Massimiliano Fabbiani[7], Ilaria Rancan[7], Mario Tumbarello[7,8], Francesca Montagnani[7,8], Claudia Sala[1], Duccio Medini[4], Raffaele De Francesco[5,6], Emanuele Montomoli[2,3,9], Rino Rappuoli[1,10,*]

[1]Monoclonal Antibody Discovery (MAD) Lab, Fondazione Toscana Life Sciences, Siena, Italy

[2]VisMederi Research S.r.l., Siena, Italy

[3]VisMederi S.r.l, Siena, Italy

[4]Data Science for Health (DaScH) Lab, Fondazione Toscana Life Sciences, Siena, Italy

[5]Department of Pharmacological and Biomolecular Sciences DiSFeB, University of Milan, Milan, Italy

[6]INGM, Istituto Nazionale Genetica Molecolare "Romeo ed Enrica Invernizzi", Milan, Italy

[7]Department of Medical Sciences, Infectious and Tropical Diseases Unit, Siena University Hospital, Siena, Italy

[8]Department of Medical Biotechnologies, University of Siena, Siena, Italy

[9]Department of Molecular and Developmental Medicine, University of Siena, Siena, Italy

[10]Department of Biotechnology, Chemistry and Pharmacy, University of Siena, Siena, Italy

[†]These authors contributed equally: Emanuele Andreano, Ida Paciello

*Corresponding author: Rino Rappuoli rino.rappuoli@biotecnopolo.it

SUPPLEMENTARY FIGURES

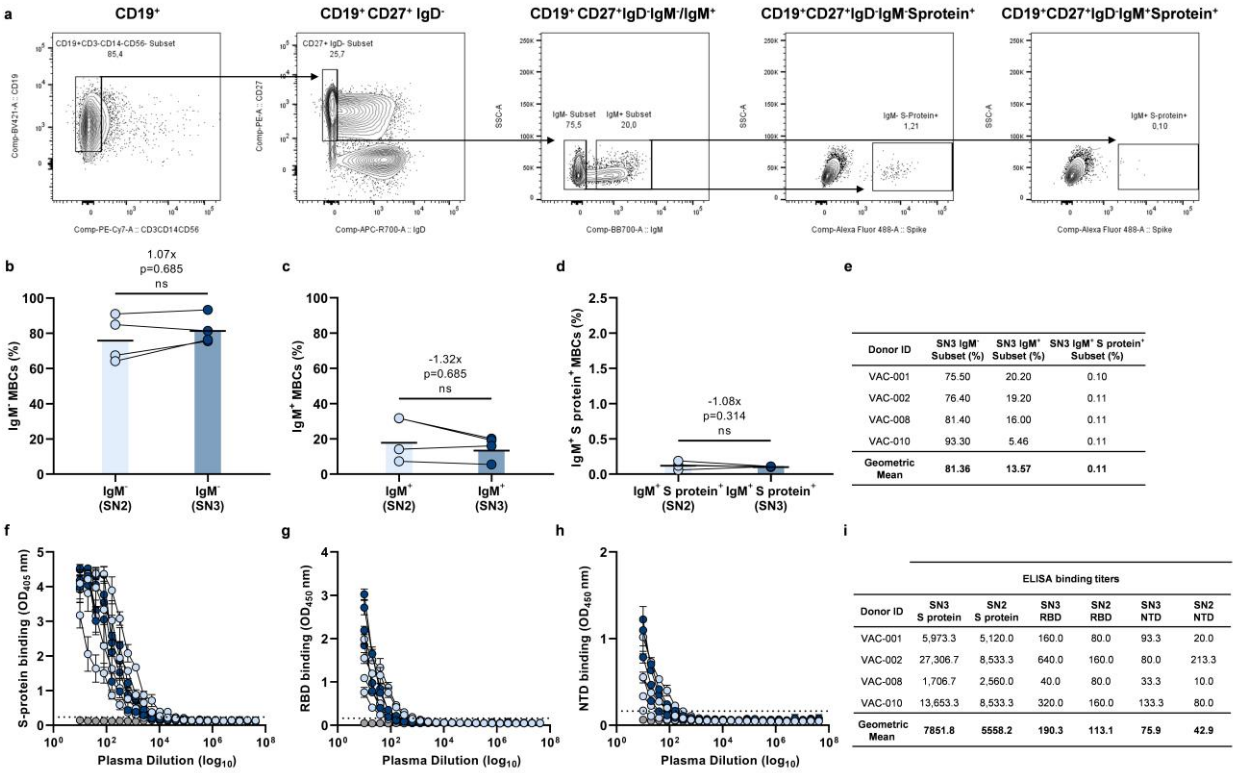

Donor ID	SN3 IgM^- Subset (%)	SN3 IgM^+ Subset (%)	SN3 IgM^+ S protein$^+$ Subset (%)
VAC-001	75.50	20.20	0.10
VAC-002	76.40	19.20	0.11
VAC-008	81.40	16.00	0.11
VAC-010	93.30	5.46	0.11
**Geometric Mean**	**81.36**	**13.57**	**0.11**

	ELISA binding titers					
Donor ID	SN3 S protein	SN2 S protein	SN3 RBD	SN2 RBD	SN3 NTD	SN2 NTD
VAC-001	5,973.3	5,120.0	160.0	80.0	93.3	20.0
VAC-002	27,306.7	8,533.3	640.0	160.0	80.0	213.3
VAC-008	1,706.7	2,560.0	40.0	80.0	33.3	10.0
VAC-010	13,653.3	8,533.3	320.0	160.0	133.3	80.0
**Geometric Mean**	**7851.8**	**5558.2**	**190.3**	**113.1**	**75.9**	**42.9**

Supplementary Fig. 1. B cell frequencies and polyclonal response. a, The gating strategy shows from left to right: $CD19^+$ B cells; $CD19^+CD27^+IgD^-$; $CD19^+CD27^+IgD^-IgM^-/IgM^+$; $CD19^+CD27^+IgD^-IgM^-Sprotein^+$; $CD19^+CD27^+IgD^-IgM^+Sprotein^+$ for one vaccinated subject (VAC-001). The same gating strategy was applied to all donors. **b-d,** The graph shows the frequency of $CD19^+CD27^+IgD^-IgM^-$ (**b**) and IgM^+ (**c**), and $CD19^+CD27^+IgD^-IgM^+$ able to bind the SARS-CoV-2 S protein trimer (S protein$^+$) (**d**) in SN2 and SN3 (n = 4 subjects/group). Black line and bars denote the geometric mean. **e,** The table summarizes the frequencies of the B cell populations for the SN3 group. **f-h**, Graphs show the ability of plasma samples from SN2 and SN3 (n = 4 subjects/group) to bind the S protein trimer (**f**), RBD (**g**) and NTD (**h**). Mean and standard deviation are denoted on each graph. Technical triplicates were performed for each experiment. **i**, The table summarizes the binding titers of each COVID-19 vaccinee and the geometric mean for SN2 and SN3. A nonparametric Mann–Whitney t test was used to evaluate statistical significances between groups. Two-tailed p-value significances are shown as *$p < 0.05$, **$p < 0.01$, ***$p < 0.001$, and ****$p < 0.0001$. Source data are provided as a Source Data file.

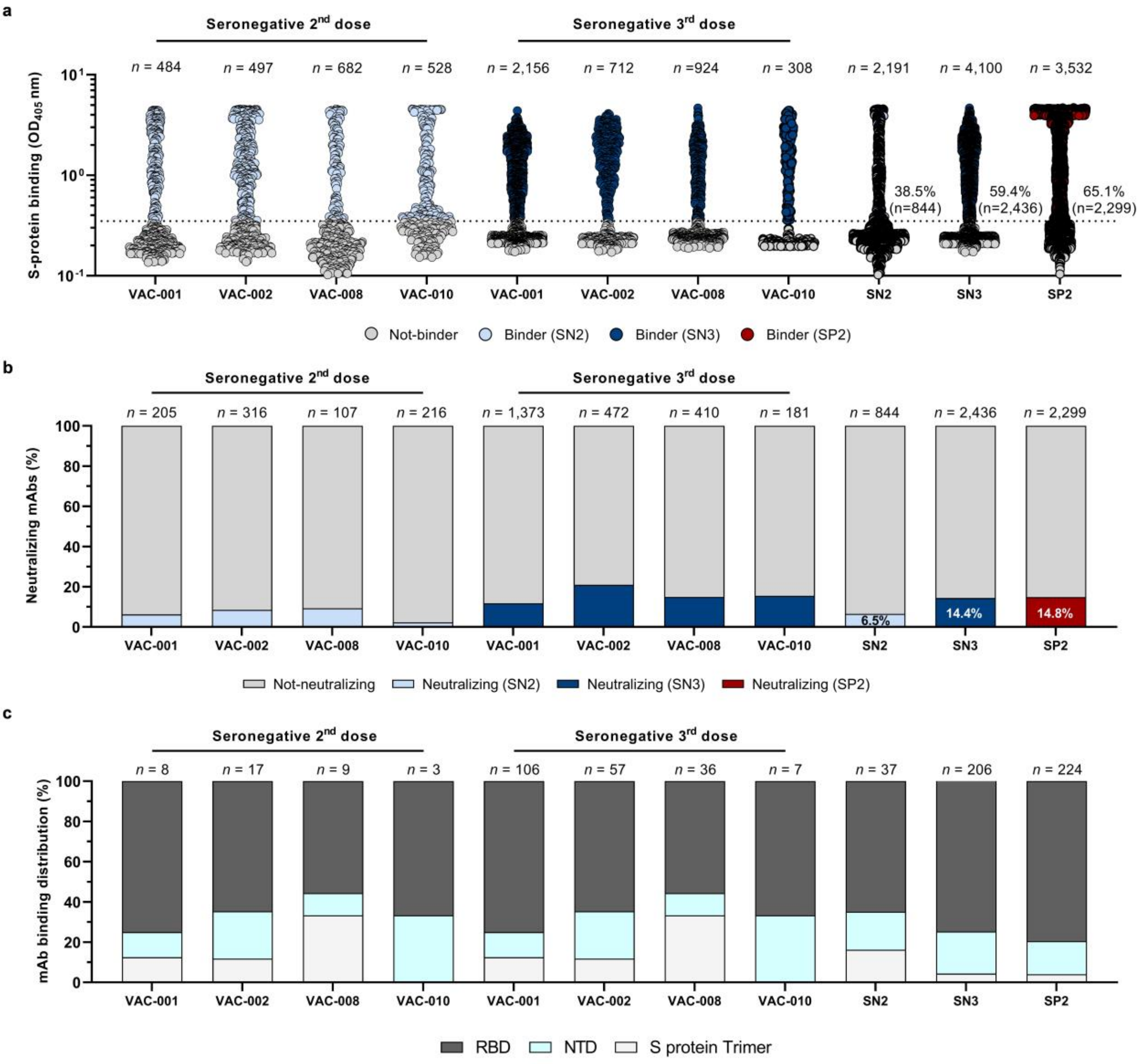

Supplementary Fig. 2. Identification of SARS-CoV-2 S protein-specific nAbs. a, The graph shows supernatants tested for binding to the Wuhan SARS-CoV-2 S protein antigen. Threshold of positivity has been set as two times the value of the blank (dotted line). Light blue, dark blue and red dots represent mAbs that bind to the S protein for SN2 and SN3, and SP2 respectively. Light gray dots represent mAbs that did not bind the S protein. **b,** The bar graph shows the percentage of not-neutralizing (gray), neutralizing mAbs from SN2 (light blue), neutralizing mAbs for SN3 (dark blue) and neutralizing mAbs for SP2 (red). The total number (*n*) of antibodies tested per individual is shown on top of each bar. **c**, The graph shows the percentage of nAbs that bind specifically the RBD (dark gray), the NTD (cyan) or that did not bind single domains but recognized exclusively the S protein in its trimetric conformation (light gray). The number (*n*) of tested nAbs per donor

is reported on top of each bar. Technical duplicates were performed for each experiment. Source data are provided as a Source Data file.

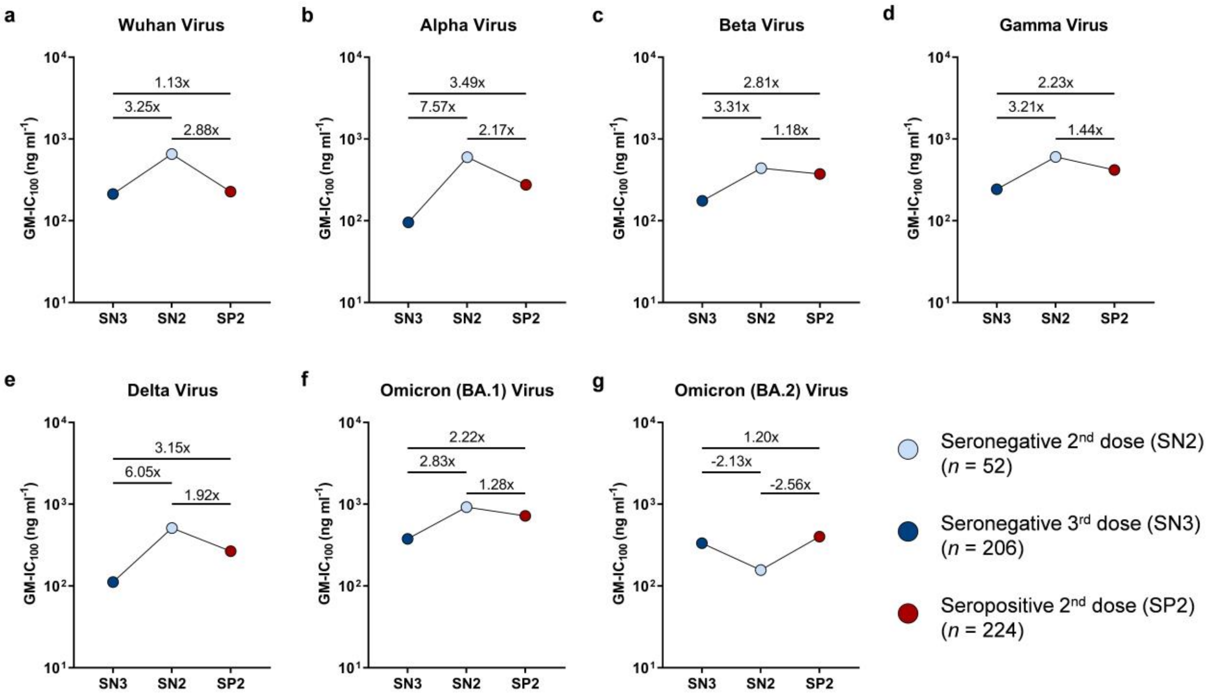

Supplementary Fig. 3. Fold-change neutralization potency against SARS-CoV-2 VoCs. a-g, The graphs show the fold-change neutralization potency shown as GM-IC_{100} (ng ml^{-1}) among SN2 (light blue), SN3 (dark blue) and SP2 (red). Fold-changes are denoted on each graph. Source data are provided as a Source Data file.

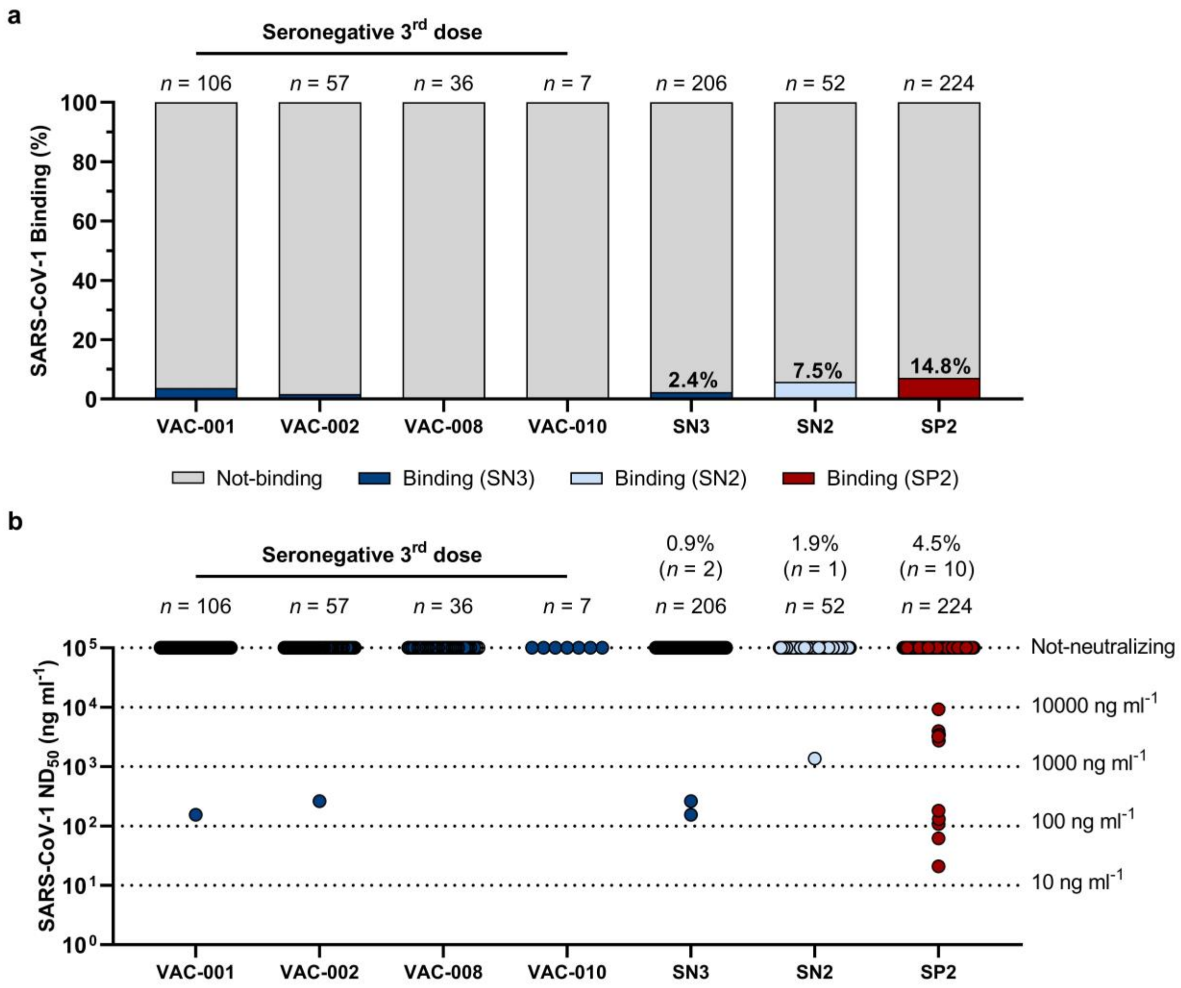

Supplementary Fig. 4. Functional characterization of SARS-CoV-1 nAbs. a, The bar graph shows the percentage of not-binding antibodies (grey), and SARS-CoV-1 binding nAbs for SN3 (dark blue), SN2 (light blue) and SP2 (red). The total number (*n*) of antibodies tested per individual is shown on the top of each bar. **b**, Dot chart shows the neutralization potency, reported as 50% neutralizing dilution (ND_{50} ng ml^{-1}), of nAbs isolated from SN3 (dark blue), SN2 (light blue) and SP2 (red). The number and percentage of nAbs from individuals who were seronegative and seropositive and neutralization ND_{50} (ng ml^{-1}) ranges (black dotted lines) are denoted on the graph. Source data are provided as a Source Data file.

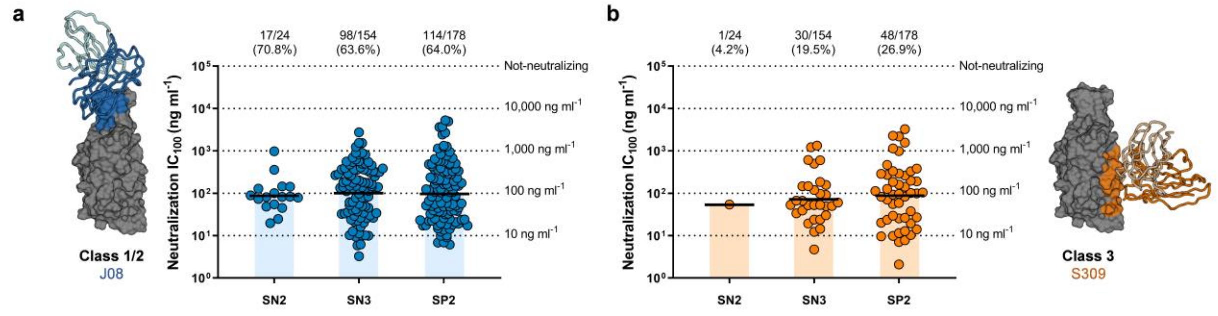

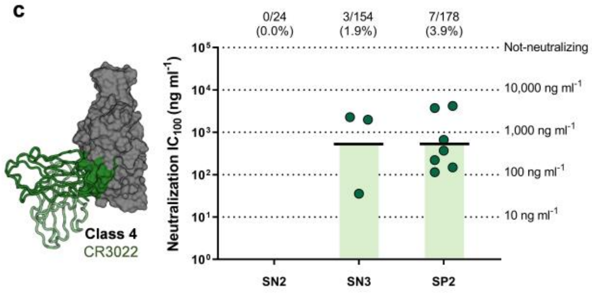

d

Cohort	Class 1/2	Class 3	Class 4
Seronegative 2nd dose (SN2) GM-IC_{100} ng ml^{-1}	89.5	54.0	N/A
Seronegative 3rd dose (SN3) GM-IC_{100} ng ml^{-1}	110.1	72.6	544.1
Seropositive 2nd dose (SP2) GM-IC_{100} ng ml^{-1}	109.5	89.5	545.9

Supplementary Fig. 5. Epitope mapping of RBD-targeting nAbs. a-c, Dot charts show the distribution of Class 1/2 (**a**), Class 3 (**b**) and Class 4 (**c**) nAbs against the original SARS-CoV-2 virus first detected in Wuhan for nAbs isolated from SN2, SN3 and SP2. The number and percentage of nAbs and neutralization IC_{100} geometric mean (black lines, light blue, orange and green bars) are denoted on each graph. **d,** the table summarizes the IC_{100} geometric mean of nAbs against Wuhan for all tested groups. Source data are provided as a Source Data file.

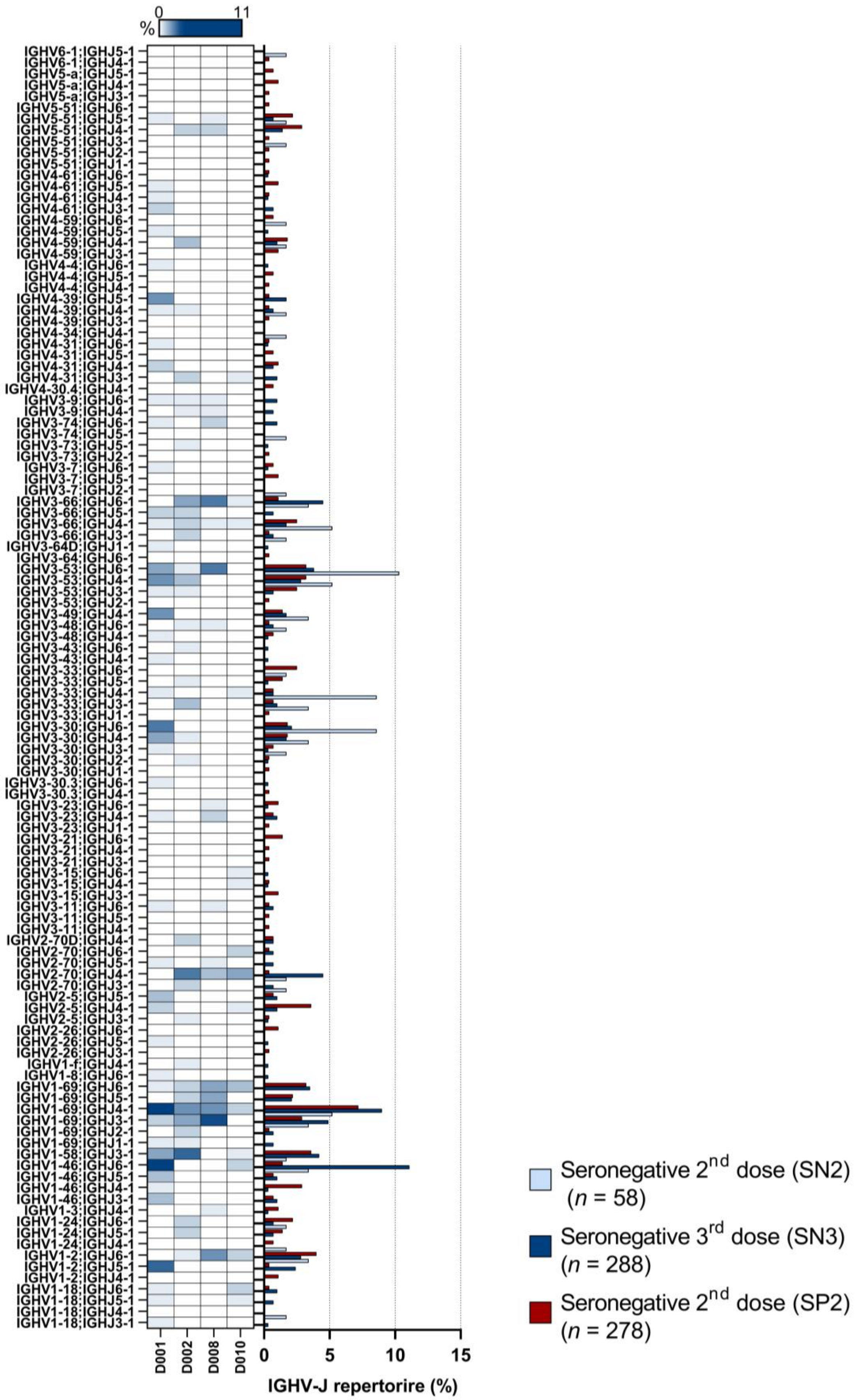

%
0
11
IGHV6-1;IGHJ5-1
IGHV6-1;IGHJ4-1
IGHV5-a;IGHJ5-1
IGHV5-a;IGHJ4-1
IGHV5-a;IGHJ3-1
IGHV5-51;IGHJ6-1
IGHV5-51;IGHJ5-1
IGHV5-51;IGHJ4-1
IGHV5-51;IGHJ3-1
IGHV5-51;IGHJ2-1
IGHV5-51;IGHJ1-1
IGHV4-61;IGHJ6-1
IGHV4-61;IGHJ5-1
IGHV4-61;IGHJ4-1
IGHV4-61;IGHJ3-1
IGHV4-59;IGHJ6-1
IGHV4-59;IGHJ5-1
IGHV4-59;IGHJ4-1
IGHV4-59;IGHJ3-1
IGHV4-4;IGHJ6-1
IGHV4-4;IGHJ5-1
IGHV4-4;IGHJ4-1
IGHV4-39;IGHJ5-1
IGHV4-39;IGHJ4-1
IGHV4-39;IGHJ3-1
IGHV4-34;IGHJ4-1
IGHV4-31;IGHJ6-1
IGHV4-31;IGHJ5-1
IGHV4-31;IGHJ4-1
IGHV4-31;IGHJ3-1
IGHV4-30.4;IGHJ4-1
IGHV3-9;IGHJ6-1
IGHV3-9;IGHJ4-1
IGHV3-74;IGHJ6-1
IGHV3-74;IGHJ5-1
IGHV3-73;IGHJ5-1
IGHV3-73;IGHJ2-1
IGHV3-7;IGHJ6-1
IGHV3-7;IGHJ5-1
IGHV3-7;IGHJ2-1
IGHV3-66;IGHJ6-1
IGHV3-66;IGHJ5-1
IGHV3-66;IGHJ4-1
IGHV3-66;IGHJ3-1
IGHV3-64D;IGHJ1-1
IGHV3-64;IGHJ6-1
IGHV3-53;IGHJ6-1
IGHV3-53;IGHJ4-1
IGHV3-53;IGHJ3-1
IGHV3-53;IGHJ2-1
IGHV3-49;IGHJ4-1
IGHV3-48;IGHJ6-1
IGHV3-48;IGHJ4-1
IGHV3-43;IGHJ6-1
IGHV3-43;IGHJ4-1
IGHV3-33;IGHJ6-1
IGHV3-33;IGHJ5-1
IGHV3-33;IGHJ4-1
IGHV3-33;IGHJ3-1
IGHV3-33;IGHJ1-1
IGHV3-30;IGHJ6-1
IGHV3-30;IGHJ4-1
IGHV3-30;IGHJ3-1
IGHV3-30;IGHJ2-1
IGHV3-30;IGHJ1-1
IGHV3-30.3;IGHJ6-1
IGHV3-30.3;IGHJ4-1
IGHV3-23;IGHJ6-1
IGHV3-23;IGHJ4-1
IGHV3-23;IGHJ1-1
IGHV3-21;IGHJ6-1
IGHV3-21;IGHJ4-1
IGHV3-21;IGHJ3-1
IGHV3-15;IGHJ6-1
IGHV3-15;IGHJ4-1
IGHV3-15;IGHJ3-1
IGHV3-11;IGHJ6-1
IGHV3-11;IGHJ5-1
IGHV3-11;IGHJ4-1
IGHV2-70D;IGHJ4-1
IGHV2-70;IGHJ6-1
IGHV2-70;IGHJ5-1
IGHV2-70;IGHJ4-1
IGHV2-70;IGHJ3-1
IGHV2-5;IGHJ5-1
IGHV2-5;IGHJ4-1
IGHV2-5;IGHJ3-1
IGHV2-26;IGHJ6-1
IGHV2-26;IGHJ5-1
IGHV2-26;IGHJ3-1
IGHV1-f;IGHJ4-1
IGHV1-8;IGHJ6-1
IGHV1-69;IGHJ6-1
IGHV1-69;IGHJ5-1
IGHV1-69;IGHJ4-1
IGHV1-69;IGHJ3-1
IGHV1-69;IGHJ2-1
IGHV1-69;IGHJ1-1
IGHV1-58;IGHJ3-1
IGHV1-46;IGHJ6-1
IGHV1-46;IGHJ5-1
IGHV1-46;IGHJ4-1
IGHV1-46;IGHJ3-1
IGHV1-3;IGHJ4-1
IGHV1-24;IGHJ6-1
IGHV1-24;IGHJ5-1
IGHV1-24;IGHJ4-1
IGHV1-2;IGHJ6-1
IGHV1-2;IGHJ5-1
IGHV1-2;IGHJ4-1
IGHV1-18;IGHJ6-1
IGHV1-18;IGHJ5-1
IGHV1-18;IGHJ4-1
IGHV1-18;IGHJ3-1
D001
D002
D008
D010
0
5
10
15
IGHV-J repertorire (%)
Seronegative 2nd dose (SN2) (n = 58)
Seronegative 3rd dose (SN3) (n = 288)
Seronegative 2nd dose (SP2) (n = 278)

Supplementary Fig. 6. Antibody germline distribution. The graph shows the IGHV;IGHJ rearrangement frequencies among SN2 (light blue), SN3 (dark blue) and SP2 (red) dose vaccinees (right panel), and the frequency within SN3 subjects (left panel). Source data are provided as a Source Data file.

SUPPLEMENTARY TABLES

Supplementary Table 1. Clinical details of seronegative 3rd dose and seropositve 2nd dose COVID-19 vaccinees.

Subject ID	Gender	Age	SARS-CoV-2 infection	First Dose (dd/mm/yy)	Second Dose (dd/mm/yy)	Third Dose (dd/mm/yy)	Vaccine	Days from Second to Third Dose	Blood Collection (dd/mm/yy)	Days from Third Dose to Blood Collection
Seronegative 3rd dose (SN3)										
VAC-001	M	39	Not-applicable	27/12/2020	18/01/2021	28/10/2021	BNT162b2	283	13/01/2022	77
VAC-002	F	39	Not-applicable	01/01/2021	22/01/2021	08/11/2021	BNT162b2	290	13/01/2022	66
VAC-008	M	44	Not-applicable	03/01/2021	24/01/2021	23/11/2021	BNT162b2	303	12/01/2022	50
VAC-010	F	52	Not-applicable	18/02/2021	11/03/2021	09/12/2021	mRNA-1273	277	20/01/2022	42
Seropositive 2nd dose (SP2)										
VAC-003	M	38	26/10/2020	08/01/2021	15/02/2021	Not-applicable	BNT162b2	Not-applicable	09/03/2021	22
VAC-004	F	25	22/10/2020	08/02/2021	01/03/2021	Not-applicable	BNT162b2	Not-applicable	09/03/2021	8
VAC-005	M	25	02/11/2020	11/01/2021	16/02/2021	Not-applicable	BNT162b2	Not-applicable	16/03/2021	28
VAC-006	F	57	24/10/2020	16/01/2021	11/02/2021	Not-applicable	BNT162b2	Not-applicable	16/03/2021	33
VAC-009	M	46	06/11/2020	20/03/2021	Not-applicable	Not-applicable	BNT162b2	Not-applicable	07/04/2021	18

Supplementary Table 2. Summary of sorted B cells and neutralizing antibodies against SARS-CoV-2 and VoCs.

Subject	S protein⁺ MBCs Sorted	S protein⁺ mAbs (%)	Wuhan - Neutralizing antibodies (%)	Expressed - Neutralizing antibodies (%)	Expressed - Neutralizing antibodies (%) Alpha	Expressed - Neutralizing antibodies (%) Beta	Expressed - Neutralizing antibodies (%) Gamma	Expressed - Neutralizing antibodies (%) Delta	Expressed - Neutralizing antibodies (%) Omicron BA.1	Expressed - Neutralizing antibodies (%) Omicron BA.2
VAC-001	2,156	1,373 (63.7)	162 (11.8)	106 (65.4)	68 (64.2)	36 (34.0)	30 (28.3)	55 (51.9)	22 (20.8)	24 (22.6)
VAC-002	712	472 (66.3)	99 (21.0)	57 (57.6)	54 (94.7)	34 (59.6)	35 (61.4)	42 (73.7)	15 (26.3)	17 (29.8)
VAC-008	924	410 (44.4)	61 (14.9)	36 (59.0)	31 (86.1)	14 (38.9)	14 (38.9)	25 (69.4)	7 (19.4)	9 (25.0)
VAC-010	308	181 (58.8)	28 (15.5)	7 (25.0)	6 (85.7)	2 (28.6)	2 (28.6)	4 (57.1)	1 (14.3)	2 (28.6)
**Total**	**4,100**	**2,436 (59.4)**	**350 (14.4)**	**206 (51.7)**	**159 (77.2)**	**86 (41.7)**	**81 (39.3)**	**126 (61.2)**	**45 (21.8)**	**52 (25.2)**
